# Guidance to applying for health research grants in the UK

**DOI:** 10.3126/nje.v12i4.50998

**Published:** 2022-12-31

**Authors:** Brijesh Sathian, Edwin van Teijlingen, Indrajit Banerjee, Russell Kabir

**Affiliations:** 1 Geriatrics and long term care department, Rumailah Hospital, Hamad Medical Corporation, Doha, Qatar; 2 Centre for Midwifery, Maternal and Perinatal Health, Bournemouth University, Bournemouth, UK; 3 Sir Seewoosagur Ramgoolam Medical College, Belle Rive, Mauritius; 4 School of Allied Health, Anglia Ruskin University, Essex, UK

A research proposal is the start and the foundation for many studies, “whether it is to conduct an academic research project or to apply for funding and support for a specific study [1]. The authors will use their experiences and insights to guide new international researchers who are considering their first research grant application in the UK. The chances of failure in applying for health research funding in the UK should not be underestimated. The typical success rate in the UK in health research varies from one in five [2] to even lower success rates.

Some funding bodies ask researchers to submit a full proposal in reply to their calls. However, many funders have a two-staged grant application system. First, anybody can submit a general short outline proposal with not too much detail, and once this initial idea has been accepted by the funding panel as having potential, in the second round selected people are invited to submit an in-depth full proposal.

Hundreds of research organizations, funding organizations, and supporting institutions make up the UK research system. These organizations differ in size, emphasis, and internal structure. *Whereas this variety is a unique strength for quality of research*, but it increases the volume and complexity of research administration, which adds to the competition to get more grants.

Researchers should understand the functions of various organizations, such as funders, authorities, academic institutions, NHS Trusts, and public and non-profit research organizations. An extensive array of research financing options is available to go along with this variety of organizations. Direct government funding, like Quality-Related (QR) funding in Wales, England, and Northern Ireland, the Research Postgraduate Grant and Research Excellence Grant in Scotland, and competitive project funding, like “responsive mode” or challenge-led funding, have very different formats and specifications.

According to the Independent Review of Research Bureaucracy - Final Report, just 20% of research funding applications are generally successful [2]. As a result, single stage processes that demand applicants to supply all the information up front result in the majority of applicants using this information unproductively and wasting it. Two step application procedures may result in system improvements, but they may also require additional time or resources from funders. UKRI and other organizations are now experimenting similar techniques.

They advised the funders to experiment with application procedures to lighten the load on applicants, such as two-stage procedures where the amount of information needed rises in proportion to the possibility of receiving funding. Funders should cooperate to enhance consistency across their application procedures, including in the language they use and the questions they pose when necessary. In the beginning, UKRI ought to assist Research Councils in this. Funders should consider what modifications to evaluation procedures may be required to account for changes to application models.

This should contain the data required for national security assessments as well as creative strategies, such testing out novel models like randomly allocating funds or using peer reviewer triage to reduce the number of applications requiring complete peer review. Funders need to make sure that the application procedures uphold their pledges to diversity, inclusion, and equality. In most cases, funders should waive the letter of support requirement from applications.

Following the COVID-19 pandemic, the UK clinical research delivery system still faces hurdles in the delivery of research. The pandemic’s continued effects on the backlog in seeing patients, manpower constraints, and the requirement to finish specific COVID-19 research have caused delays in the completion of several studies. As a result, fewer studies are now able to successfully recruit participants and finish on time.

Workload, staffing, and the need to clear the elective backlog due to the combined effects of chronic underfunding and Brexit continue to put strain on the NHS’s ability to support research delivery. Some studies have difficulty in the present context and have limited likelihood of achieving their study endpoints and objectives due to a lack of resources and capacity. For others, the lack of resources and capability makes it difficult to execute studies within reasonable timeframes. The research system must prioritize studies that can be completed given the capability and resources at its disposal, while acknowledging that some studies (such as those involving rare disorders) may require less frequent recruitment of participants.

The Department of Health and Social Care (DHSC) launched the Research Reset programme in response to the ongoing challenges in research delivery with the goal of making portfolio delivery attainable within anticipated timelines (time and target) and sustainable within the resource and capability we currently have in the NHS [3]. By working with funders and sponsors to promote the evaluation of studies that have already finished or that are unlikely to be able to fulfill their endpoints in the present climate, it seeks to free up capacity throughout the research system.

The programme is overseen by the DHSC with input from an advisory group made up of representatives from medical research charities, industry, NHS Research and Development, research delivery workforce representatives across NHS settings, patient and public representatives, universities, the Royal Colleges, the Medical Research Council (MRC), and NHS regions throughout the UK.

According to the present standards, NIHR will continue to add new research to the portfolio throughout the Research Reset programme.

Building on NIHR achievements and the lessons learned in response to COVID-19, the future of clinical research delivery outlines the vision for a clinical research environment that is more patient-centered, pro-innovation, and digitally enabled. It also aims to maximize the UK’s capacity to profit from cutting-edge innovations across all treatments and technologies, all research phases, and all conditions [4]. Five key themes underpin the NIHR’s vision are (1) clinical research embedded in the NHS, (2) patient-centred research, (3) streamlined, efficient and innovative research, (4) research enabled by data and digital tools, and (5) a sustainable and supported research workforce.

There have been efforts in the UK to bring various government departments and research funders working in international development together in the UK Collaborative on Development Research (UKCDR). Which is why we have listed in it separately from several of its constituents in Table 1.

## Two case studies

We offer two case studies to illustrate some of the issues that may occur in applying for grants from Uk organisations. Both examples relate to research conducted in Nepal. The first example is a three-year project is UK-funded under the Health Systems Research Initiative. This study ‘the impact of federalisation on Nepal’s health system: a longitudinal analysis’ is a collaboration between researchers at the University of Sheffield, Bournemouth University and the University of Huddersfield in the UK and Manmohan Memorial Institute of Health Science and PHASE Nepal both in Kathmandu [5-7]. We first applied for funding in 2018, the committee seemed to like the research idea but were worried about the potential slow progress of the federaisation process in Nepal, the lack academic experts in Nepal and the large proportion of the cost related to the UK. In the 2019 we addressed these issues, obviously to the funders’ satisfaction as we were successful in our resubmission.

The second case study centres on an application for a DelPHE (Round 4), British Council award [8-9]. Our study ‘Partnership on Improving Access to Research Literature for HE Institutions in Nepal’ (PARI Initiative) was a collaboration between Tribhuvan University (TU), the oldest and largest university in Nepal, and the University of Aberdeen and Bournemouth University in the UK. Our initial application for DelPHE funding with Stupa College in Kathmandu, a smallish not-for-profit college, was unsuccessful. In the feedback from the funder we were advised to collaborate with a larger university, preferably a government university to increase the chance that our intervention/training would be incorporated in future training and curricula. Therefore, we submitted a similar application the next year with a new partner in Nepal, namely the Central Department of Population Studies at Tribhuvan University (TU). This is resubmission was then accepted and funded.

Always consider collaborating with someone in the home country of the funding agency, in this case with an academic at a UK university. Both getting through the first round of a two-staged application and being asked to resubmit means you have had a dummy run. Submitting after feedback from experts on a funding board/panel or from bureaucrats working for the funder offers you the opportunity to improve the application. The improvements can be methodologically, or in the detail of some of the application of your prosed methods, of financially, or ethically, etc. In other words, you can fine tune your new submission and home in on the issues the funders find important. MRC provides 37-page document *Guidance for Peer Reviewers* [10].

An innovative and competitive grant proposal will have greater odds to get success. Making connections with researchers with similar research interest to collaborate can help increase your changes of getting funding. Looking for funding opportunities in your own field of expertise or that of your collaboratiors can assist to make sure that the application and the funder are a suitable fit. It is crucial to thoroughly review funding agencies’ focus because funding requirements and eligibility requirements might differ greatly between organisations, but also from year to year for the same funder. The funder’s past awards may serve as a useful indicator of the kinds of studies they are likely to support and the details of winning proposals.

## Figures and Tables

**Table 1:** Key funding agencies in the UK

Organisation	Focus*	Website
**NIHR**	Public health, clinical evaluation, translation, social care	https://www.nihr.ac.uk/
**Wellcome Trust**	Mental health, infectious disease, climate and health	https://wellcome.org/
**Economic & Social Research Council (ESRC)**	economic, social, behavioural and human data science	https://www.ukri.org/councils/esrc/
**Medical Research Council (MRC)**	Funds research to prevent illness, develop therapies & improve human health	https://www.ukri.org/councils/mrc/
**Arts & Humanities Research Council (AHRC)**	Funds outstanding original research across the whole range of the arts and humanities	https://www.ukri.org/councils/ahrc/
**Biotechnology & Biological Sciences Research Council (BBSRC)**	Committee A: animal disease, health & welfare Committee B: plants, microbes, food & sustainability Committee C: genes, development & STEM approaches to biology. Committee D: molecules, cells & biotechnology.	https://www.ukri.org/councils/bbsrc/
**Engineering & Physical Sciences Research Council (EPSRC)**	Engineering and physical sciences for UK capability to benefit society and the economy	https://www.ukri.org/councils/epsrc/
**Innovate UK**	New approaches in technologies incl. manufacturing, artificial intelligence, digital, electronics, sensors, biosciences, quantum, and advanced computing	https://www.ukri.org/councils/innovate-uk/
**Natural Environment Research Council (NERC)**	Environmental science	https://www.ukri.org/councils/nerc/
**UK Collaborative on Development Research (UKCDR)**	provide overview of latest international development research funding opportunities in UK	https://www.ukcdr.org.uk/funding-landscape/funding-calls/
**Science & Technology Facilities Council (STFC)**	fundamental research in astronomy, physics and space science	https://www.ukri.org/councils/stfc/
**Cancer Research UK**	Working with civil societies and researcher around the world to grow policy evidence base for regional, national or local change that will prevent cancer incidence in low- and middle- income countries Bring together world’s best minds to tackle cancer’s biggest challenges. Collaborate across disciplines and continents to research cancer.	https://www.cancerresearchuk.org/

* Relevant to low- and middle-income countries
